# Simultaneous activation of CXC chemokine receptor 4 and histamine receptor H1 enhances calcium signaling and cancer cell migration

**DOI:** 10.1038/s41598-023-28531-1

**Published:** 2023-02-02

**Authors:** Chulo Park, Jin-Woo Lee, Kiheon Kim, Dong-Seung Seen, Jae-Yeon Jeong, Won-Ki Huh

**Affiliations:** 1 School of Biological Sciences, Seoul National University, Seoul, 08826 Republic of Korea; 2 GPCR Therapeutics Inc., Gwanak-gu, Seoul, 08790 Republic of Korea; 3 Institute of Microbiology, Seoul National University, Seoul, 08826 Republic of Korea

**Keywords:** Target identification, Cell migration, Cell signalling

## Abstract

C-X-C chemokine receptor 4 (CXCR4) is widely overexpressed in various types of cancer and is involved in several cancer phenotypes including tumor growth, survival, and metastasis. The roles of histamine and histamine receptor H1 (HRH1) in cancer pathogenesis remain controversial. Here, we show that HRH1 is widely expressed in various cancer cell lines and cancer tissues and that coexpression of CXCR4 and HRH1 is associated with poor prognosis in breast cancer. Using bimolecular fluorescence complementation and bioluminescence resonance energy transfer donor saturation assays, we demonstrate that CXCR4 and HRH1 can assemble into a heteromeric complex. Simultaneous activation of CXCR4 and HRH1 synergistically increases calcium flux in MDA-MB-231 cells that endogenously express CXCR4 and HRH1 but not in cells deficient in CXCR4 or HRH1. Costimulation of CXCR4 and HRH1 also significantly enhances CXCL12-induced MDA-MB-231 cell migration, while histamine alone does not induce cell migration. Synergistic effects on calcium flux and cell migration are inhibited by the Gα_i_ inhibitor pertussis toxin and the Gα_q_ inhibitor YM254890, suggesting that the Gα_i_ and Gα_q_ pathways are involved in the synergy. Enhanced calcium signaling and cell migration are also observed in NCI-H23 and HeLa cells, which coexpress CXCR4 and HRH1. Taken together, our findings demonstrate an interplay between CXCR4 and HRH1, and suggest the possibility of the CXCR4-HRH1 heteromer as a potential therapeutic target for anticancer therapy.

## Introduction

G protein-coupled receptors (GPCRs) are the largest family of plasma membrane receptors and mediate most cellular responses to extracellular stimuli, including hormones, neurotransmitters, light, odors, and taste^1^. Because GPCRs regulate most physiological responses, they are involved in several pathological pathways. Indeed, over 34% of FDA-approved drugs target GPCRs^2–4^. GPCRs are also important in cancer, as several GPCRs are known to be responsible for tumor growth, survival, and metastasis^5^.

CXC chemokine receptor 4 (CXCR4) is a chemokine receptor expressed on immune cells that regulates immune cell homing to the bone marrow^6,7^. CXCR4 is also responsible for breast cancer metastasis to organs that express high levels of its ligand CXCL12, such as lymph nodes, bone marrow, lung, and liver^8^. CXCR4 is widely overexpressed in at least 20 types of cancer, including breast cancer^9^, prostate cancer^10^, melanoma^11^, and neuroblastoma^12^. CXCR4 plays important roles in tumor growth^13^, angiogenesis^14^, metastasis^15,16^, and therapeutic resistance^17^. Overexpression of CXCR4 is associated with poor prognosis in various cancers such as breast, lung, and colorectal cancers^18–20^. CXCL12 is also constitutively expressed by cancer-associated fibroblasts in the tumor microenvironment^14^, and paracrine signaling between cancer-associated fibroblasts and CXCR4-expressing tumor cells regulates tumor survival and metastasis^14,21^. Previous studies have shown that CXCR4 function can be regulated through heteromerization with other GPCRs. Cannabinoid receptor type 2 (CB2) inhibits CXCR4-mediated migration through heteromerization with CXCR4 and downregulation of Gα_13_/RhoA signaling^22,23^. CXCR7 forms heteromers with CXCR4 and enhances CXCL12-mediated cell migration through the β-arrestin and ERK pathways^24^.

Histamine is a biological amine mainly produced by tissue mast cells in response to allergic stimuli^25^. Histamine triggers allergic reactions, such as vasodilation, increased vascular permeability, bronchoconstriction, and gastric acid secretion, through four types of GPCRs named histamine receptor H1 (HRH1), HRH2, HRH3, and HRH4. HRH1 is ubiquitously expressed and is responsible for classical allergic reactions. HRH2 is highly expressed in immune cells and digestive organs, and is important for immune functions and gastric acid secretion. HRH3 is exclusively expressed in neurons, and HRH4 is mainly expressed in immune cells. Lung, breast, endometrial, colorectal, and melanoma skin cancers exhibit increased levels of histamine and histidine decarboxylase, an enzyme responsible for histamine production^26–31^. However, histamine treatment produces controversial results in cancer cell proliferation, survival, and migration, depending on the concentrations of histamine and types of histamine receptors^31–33^.

In the present study, we show that HRH1 is the major histamine receptor highly expressed in many cancer cell lines and cancer tissues and that coexpression of CXCR4 and HRH1 is associated with lower survival in breast cancer by analyzing publicly available RNA-seq databases. We demonstrate that CXCR4 physically interacts with HRH1 using bimolecular fluorescence complementation (BiFC) and bioluminescence resonance energy transfer (BRET) donor saturation assays. Costimulation of CXCR4 and HRH1 synergistically increases calcium flux and CXCL12-induced cell migration in various cancer cells that express CXCR4 and HRH1 endogenously. Using the Gα_i_ inhibitor pertussis toxin and the Gα_q_ inhibitor YM254890, we show that the Gα_i_ and Gα_q_ pathways are involved in the synergistic effects on calcium flux and cell migration. Our results suggest the possibility of the CXCR4-HRH1 heteromer as a potential therapeutic target for anticancer therapy.

## Results and discussion

### Coexpression of CXCR4 and HRH1 in breast cancer correlates with poor patient prognosis

**Figure 1:**
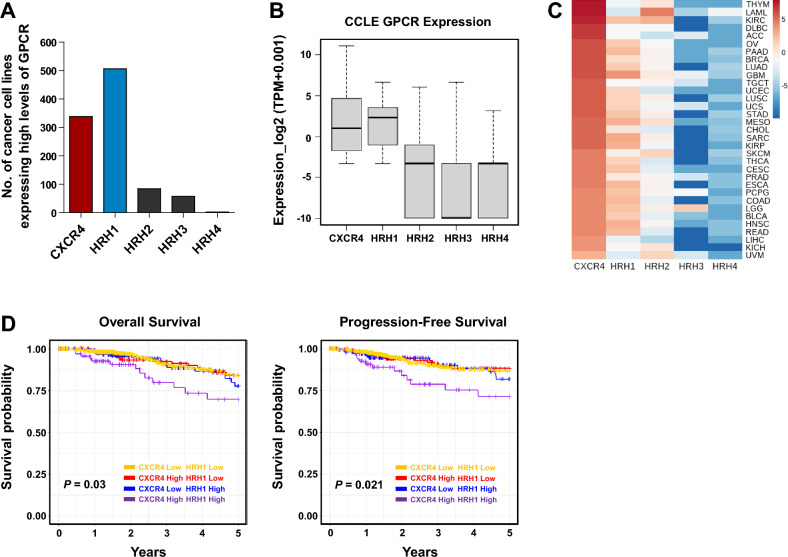
Coexpression of CXCR4 and HRH1 in breast cancers correlates with poor patient prognosis. (**A**) Expression of CXCR4 and histamine receptor subtypes in cancer cell lines based on RNA-seq data from the CCLE. Cancer cell lines expressing CXCR4, HRH1, HRH2, HRH3, and HRH4 mRNAs higher than 5 TPM were counted. (**B**) Box plots of the expression levels of CXCR4 and histamine receptors in cancer cell lines from the CCLE. (**C**) Heatmap analysis of the expression of CXCR4 and histamine receptors in 33 TCGA cancer types. The median log2 (TPM + 0.001) values are shown with the scale bar. *ACC* adrenocortical cancer, *BLCA* bladder urothelial carcinoma, *BRCA* breast invasive carcinoma, *CESC* cervical and endocervical cancer, *CHOL* cholangiocarcinoma, *COAD* colon adenocarcinoma, *DLBC* diffuse large B-cell lymphoma, *ESCA* esophageal carcinoma, *GBM* glioblastoma multiforme, *HNSC* head and neck squamous cell carcinoma, *KICH* kidney chromophobe, *KIRC* kidney clear cell carcinoma, *KIRP* kidney papillary cell carcinoma, *LAML* acute myeloid leukemia, *LGG* brain lower grade glioma, *LIHC* liver hepatocellular carcinoma, *LUAD* lung adenocarcinoma, *LUSC* lung squamous cell carcinoma, *MESO* mesothelioma, *OV* ovarian serous cystadenocarcinoma, *PAAD* pancreatic adenocarcinoma, *PCPG* pheochromocytoma and paraganglioma, *PRAD* prostate adenocarcinoma, *READ* rectum adenocarcinoma, *SARC* sarcoma, *SKCM* skin cutaneous melanoma, *STAD* stomach adenocarcinoma, *TGCT* testicular germ cell tumor, *THCA* thyroid carcinoma, *THYM* thymoma, *UCEC* uterine corpus endometrioid carcinoma, *UCS* uterine carcinosarcoma, *UVM* uveal melanoma. (**D**) Coexpression of CXCR4 and HRH1 reduces overall survival and progression-free survival in breast cancer. Kaplan–Meier survival curves were analyzed using clinical data from TCGA Breast Invasive Carcinoma. Patients were divided into four groups according to the expression of CXCR4 and HRH1, and comparisons between groups were performed by the log-rank test. Pooled log-rank *P* values are presented for overall survival (left panel) and progression-free survival (right panel).

We first investigated the expression of CXCR4 and HRH1 in cancer. Analysis of publicly available cancer cell line RNA-seq data sourced from the Cancer Cell Line Encyclopedia (CCLE)^34^ showed that CXCR4 and HRH1 were expressed in 36.2% and 54.2% of 934 cancer cell lines, respectively (Fig. 1A), and that HRH1 was the most frequently and highly expressed histamine receptor of the four HRHs (Fig. 1B). CXCR4 and HRH1 were coexpressed in cell lines from neuroendocrine origin, such as astrocytoma, glioblastoma, medulloblastoma, and neuroblastoma, and in those from other solid cancers, such as bile duct cancer, breast cancer, cecum and colon cancer, gastric cancer, hepatic cancer, mesothelioma, ovarian cancer, pancreatic cancer, renal cancer, thyroid cancer, and uterine cancer (Table S1). CXCR4 and HRH1 were not coexpressed in colorectal cancer, melanoma, multiple myeloma, and prostate cancer cell lines. According to RNA-seq data from The Cancer Genome Atlas (TCGA), CXCR4 was also highly coexpressed with HRH1 in various cancers, such as glioblastoma, renal cancer, mesothelioma, sarcoma, breast cancer, and pancreatic cancer (Fig. 1C). We next investigated the association between coexpression of CXCR4 and HRH1 in cancer and patient prognosis. Kaplan‒Meier plots showed that breast cancer patients with high CXCR4 and HRH1 expression had reduced overall survival and progression-free survival compared with other groups (Fig. 1D). These results raise the possibility that coexpression of CXCR4 and HRH1 is associated with breast cancer progression.

### CXCR4 physically interacts with HRH1

**Figure 2:**
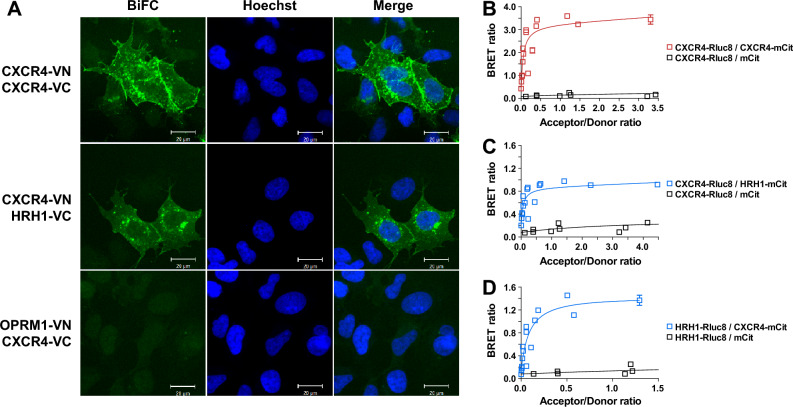
HRH1 physically interacts with CXCR4. (**A**) Analysis of CXCR4-HRH1 heteromerization using the BiFC assay. HEK293A cells were transfected with CXCR4-VN and CXCR4-VC (upper panel), CXCR4-VN and HRH1-VC (middle panel), or OPRM1-VN and CXCR4-VC (lower panel), and BiFC signals were visualized. Cell nuclei were stained with Hoechst 33342. Images are representative of three independent experiments. (**B**–**D**) Analysis of CXCR4-HRH1 heteromerization using the BRET donor saturation assay. BRET donor saturation curves of CXCR4 homomer (**B**) and CXCR4-HRH1 heteromer (**C**,**D**) were obtained with HEK293A cells transfected with a fixed amount of donor (GPCR-Rluc8) and increasing amounts of acceptor (GPCR-mCitrine) plasmids. BRET values were plotted as a function of mCitrine/Rluc8. The curves were fitted using a nonlinear regression equation assuming a single binding site and represent three independent experiments.

To understand the potential crosstalk between CXCR4 and HRH1, we examined whether CXCR4 physically interacts with HRH1 using the BiFC assay^35^. In the BiFC assay, a fluorescent protein is split into two fragments that cannot fluoresce themselves, and each fragment is tagged to the target protein. If two target proteins come into close proximity by physical interaction, then the two tagged fluorescent fragments also become close to each other, and the interaction of the target proteins can be detected by the recovered fluorescence. Consistent with previous reports^36,37^, coexpression of CXCR4-VN and CXCR4-VC in HEK293A cells resulted in robust BiFC signals at the plasma membrane and cytoplasm, implying the presence of CXCR4 homomers (Fig. 2A, upper panel). Remarkably, BiFC signals were also observed in cells coexpressing CXCR4-VN and HRH1-VC (Fig. 2A, middle panel) or CXCR4-VC and HRH1-VN (Fig. S1A), suggesting that CXCR4 forms heteromers with HRH1. In contrast, BiFC signals were not observed in cells coexpressing CXCR4 and opioid receptor μ type 1 (OPRM1) (Fig. 2A, lower panel), although OPRM1 was expressed well on the cell surface as visualized by antibody staining (Fig. S1B) and flow cytometry (Fig. S1C). These results suggest that CXCR4 physically interacts with HRH1 and that the interaction between CXCR4 and HRH1 is specific.

To further validate the CXCR4-HRH1 interaction, we performed a BRET donor saturation assay^38,39^. In the BRET donor saturation assay, a fixed concentration of bioluminescent-tagged donor protein and variable concentrations of fluorescent-tagged acceptor protein are expressed in cells. The interaction between two proteins can be examined by quantifying the dependence of the BRET signal on the acceptor/donor expression ratio. Cells expressing increasing concentrations of CXCR4-mCitrine against a fixed amount of CXCR4-Rluc8 exhibited hyperbolic increases in the BRET ratios (Fig. 2B), indicating the formation of CXCR4 homomers. When cells were transfected with increasing amounts of HRH1-mCitrine against a fixed amount of CXCR4-Rluc8, hyperbolic increases in the BRET ratios were obtained (Fig. 2C). Increasing amounts of CXCR4-mCitrine against a fixed amount of HRH1-Rluc8 also led to hyperbolic increases in the BRET ratios (Fig. 2D). In contrast, cells expressing increasing amounts of mCitrine with a fixed amount of CXCR4-Rluc8 or HRH1-Rluc8 showed linear increases in the BRET ratios (Fig. 2B–D), which are indicative of nonspecific random collisions. These results suggest that CXCR4 forms heteromers with HRH1 when they are coexpressed. To our knowledge, this is the first report of CXCR4-HRH1 heteromerization.

### CXCR4 and HRH1 are endogenously coexpressed in the breast cancer cell line MDA-MB-231

**Figure 3:**
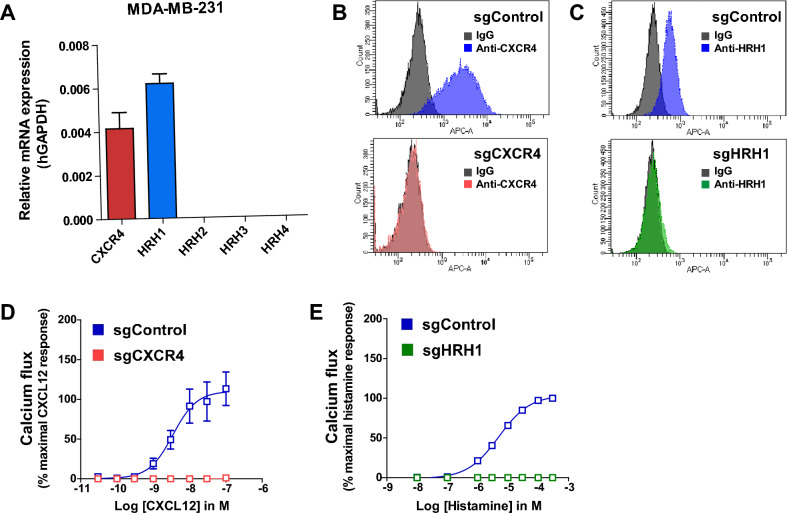
CXCR4 and HRH1 are endogenously coexpressed in MDA-MB-231 breast cancer cells. (**A**) Analysis of the mRNA expression of CXCR4 and histamine receptors in MDA-MB-231 cells using RT‒qPCR. Endogenous β-actin was used to normalize the expression of target genes. (**B**,**C**) Expression of CXCR4 and HRH1 proteins in MDA-MB-231 cells. Cells were transduced with lentiviruses encoding CRISPR/Cas9 and nontargeting single guide RNA (sgControl), CXCR4-targeting single guide RNA (sgCXCR4), or HRH1-targeting single guide RNA (sgHRH1). After puromycin selection, cells were stained with an anti-CXCR4 antibody (**B**) or an anti-HRH1 antibody (**C**). (**D**,**E**) Functional expression of CXCR4 and HRH1 in MDA-MB-231 cells. Cells were stimulated with increasing doses of CXCL12 (**D**) or histamine (**E**), and calcium flux was measured. Data are expressed as mean ± s.e.m. (n = 3).

To investigate the role of the CXCR4-HRH1 heteromer in cancer cells, we examined the endogenous expression of CXCR4 and histamine receptors in MDA-MB-231 breast cancer cells using real-time quantitative PCR (RT‒qPCR). Significant levels of CXCR4 and HRH1 mRNAs were detected in MDA-MB-231 cells, while mRNAs for other histamine receptors were undetectable (Fig. 3A). In addition, histamine-induced calcium flux was completely reduced by the HRH1-selective antagonist pyrilamine but not by the HRH2-selective antagonist ranitidine, the HRH3-selective antagonist pitolisant, or the HRH4-selective antagonist JNJ-7777120 (Fig. S2). These results suggest that HRH1 is the main histamine receptor responsible for histamine-induced calcium flux in MDA-MB-231 cells.

Next, we investigated whether CXCR4 and HRH1 proteins are expressed in MDA-MB-231 cells. CXCR4 expression on the surface of MDA-MB-231 cells was successfully detected by flow cytometry using an anti-CXCR4 antibody (Fig. 3B, upper panel). When the CXCR4 gene was deleted by CRISPR/Cas9, the surface expression of CXCR4 was completely lost (Fig. 3B, lower panel). Flow cytometry using an anti-HRH1 antibody also demonstrated that HRH1 was expressed in MDA-MB-231 cells (Fig. 3C, upper panel) and that its expression was lost upon deletion of HRH1 by CRISPR/Cas9 (Fig. 3C, lower panel). Consistent with these observations, CXCL12 failed to induce calcium flux in CXCR4-deficient MDA-MB-231 cells (Fig. 3D) and histamine-induced calcium flux was also lost in HRH1-deficient MDA-MB-231 cells (Fig. 3E). These results indicate that CXCR4 and HRH1 are endogenously expressed in MDA-MB-231 cells and mediate proper calcium mobilization.

### Costimulation of CXCR4 and HRH1 induces synergistic calcium flux in MDA-MB-231 cells

**Figure 4:**
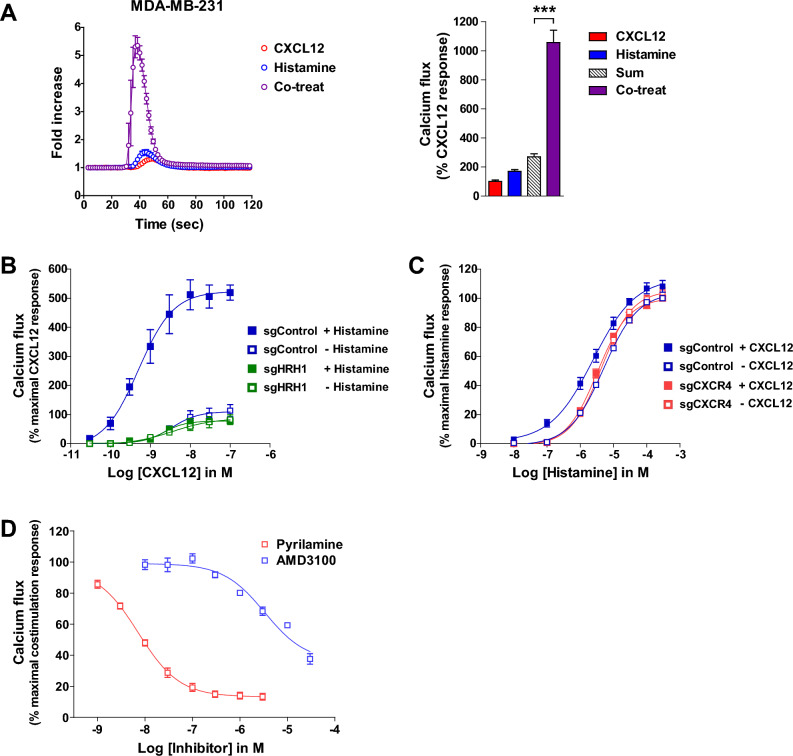
Costimulation of CXCR4 and HRH1 induces synergistic calcium mobilization in MDA-MB-231 cells. (**A**) MDA-MB-231 cells were stimulated with 3 nM CXCL12, 100 nM histamine, or both agonists at the same concentrations, and intracellular calcium flux was measured (left panel). The area under the curve of each calcium response was calculated and normalized to that of CXCL12 (right panel). Data are expressed as mean ± s.e.m. (n = 3). ***P < 0.001. (**B**,**C**) Synergistic calcium flux is mediated by CXCR4 and HRH1. Intracellular calcium flux was measured by stimulating HRH1 knockout (sgHRH1) or control (sgControl) cells with increasing concentrations of CXCL12 in the absence or presence of 100 nM histamine (**B**). Intracellular calcium flux was measured by stimulating CXCR4 knockout (sgCXCR4) or control (sgControl) cells with increasing concentrations of histamine in the absence or presence of 3 nM CXCL12 (**C**). Data are expressed as mean ± s.e.m. (n = 3–6). (**D**) Inhibition of the calcium response by the CXCR4 antagonist AMD3100 and the HRH1 antagonist pyrilamine. MDA-MB-231 cells were pretreated with increasing concentrations of AMD3100 or pyrilamine, and costimulated with 3 nM CXCL12 and 100 nM histamine. Data are expressed as mean ± s.e.m. (n = 3).

It has been reported that CXCR4-mediated calcium signaling regulates cell migration and survival^40–42^. HRH1 also induces calcium signaling via Gα_q_ protein and phospholipase C (PLC)^43,44^. To investigate whether calcium signaling induced by CXCR4 is affected by HRH1 or vice versa, MDA-MB-231 cells were stimulated with CXCL12 and histamine, and intracellular calcium flux was measured. Interestingly, calcium flux induced by simultaneous treatment with CXCL12 and histamine was significantly higher than the sum of calcium flux induced by CXCL12 and histamine alone (Fig. 4A). The addition of histamine markedly increased CXCL12-induced calcium flux at all CXCL12 doses in control cells, while this increase was not observed in cells deficient in HRH1 (Fig. 4B) or CXCR4 (Fig. S3A). Similarly, the addition of CXCL12 slightly increased histamine-induced calcium flux in control cells but not in cells deficient in CXCR4 (Fig. 4C) or HRH1 (Fig. S3B). Consistent with these observations, the CXCR4 antagonist AMD3100 and the HRH1 antagonist pyrilamine abolished calcium flux in MDA-MB-231 cells in a dose-dependent manner (Fig. 4D). Taken together, these results suggest that both CXCR4 and HRH1 are important for the synergistic calcium flux induced by CXCL12 and histamine cotreatment. We also examined whether HRH1 affects CXCR4-mediated Gα_i/o_ signaling, which is another major downstream signaling pathway of CXCR4. As expected, forskolin-induced cAMP production was reduced by CXCL12 (Fig. S4). However, the addition of histamine did not affect CXCR4-mediated cAMP responses. This result suggests that HRH1 affects CXCR4-mediated calcium signaling but not cAMP signaling.

### Both the Gα_i/o_ and Gα_q/11_ pathways are important for synergistic calcium flux induced by costimulation of CXCR4 and HRH1

**Figure 5:**
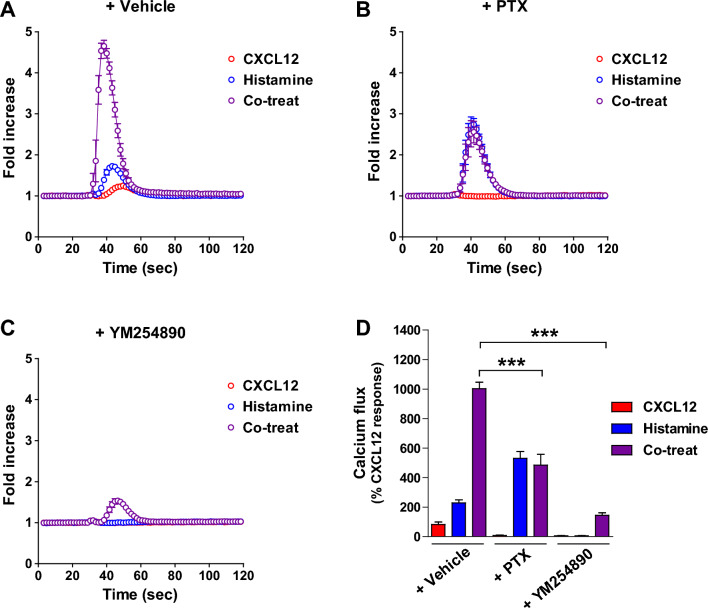
Activation of both Gα_i/o_ and Gα_q/11_ is important for synergistic calcium mobilization induced by costimulation of CXCR4 and HRH1. MDA-MB-231 cells were pretreated with vehicle (**A**), PTX (100 ng/ml, overnight) (**B**), or YM254890 (300 nM, 30 min) (**C**) prior to agonist treatment. Cells were stimulated with 3 nM CXCL12, 100 nM histamine, or both, and intracellular calcium flux was measured. (**D**) The area under the curve of each calcium response was calculated and normalized to that of CXCL12. Data are expressed as mean ± s.e.m. (n = 3). ***P < 0.001.

To understand the signaling pathways that regulate synergistic calcium flux upon costimulation of CXCR4 and HRH1, MDA-MB-231 cells were treated with inhibitors that block Gα activation. Pretreatment with vehicle alone had no effect on CXCL12-histamine-induced synergistic calcium mobilization (Fig. 5A). Consistent with previous reports that the Gα_i/o_ inhibitor pertussis toxin (PTX) inhibits CXCL12/CXCR4-mediated calcium signaling^45,46^, pretreatment with PTX completely blocked CXCL12-induced calcium flux, while it did not affect histamine-induced calcium flux (Fig. 5B). Notably, PTX pretreatment abolished the synergistic increase in calcium flux induced by CXCL12 and histamine cotreatment, suggesting that Gα_i/o_ activation is essential not only for CXCL12-induced calcium mobilization but also for CXCL12-histamine-induced synergistic calcium flux. When cells were pretreated with the Gα_q/11_ inhibitor YM254890, both CXCL12- and histamine-induced calcium fluxes were inhibited (Fig. 5C), suggesting that Gα_q/11_ activation is crucial for CXCR4- and HRH1-mediated calcium signaling. CXCL12-histamine-induced synergistic calcium mobilization was also considerably reduced by YM254890 pretreatment. Given the above results showing that synergistic calcium flux induced by CXCL12 and histamine cotreatment was abolished by PTX and YM254890 (Fig. 5B–D), it is likely that activation of both Gα_i/o_ and Gα_q/11_ is important for increased calcium signaling upon costimulation of CXCR4 and HRH1. Consistent with our results, Pfeil et al.^47^ have recently reported that activation of both the Gα_i_ and Gα_q_ pathways results in synergistic calcium signaling; when Gα_i_- and Gα_q_-coupled GPCRs are coactivated, the occluded catalytic site of PLCβ is freed by Gα_q_, leading to amplification of calcium signaling mediated by Gβγ-PLCβ. Whether the physical interaction between CXCR4 and HRH1 affects synergistic calcium flux due to the signaling crosstalk between the Gα_i_ and Gα_q_ pathways is not yet clear and will need further investigation.

### Costimulation of CXCR4 and HRH1 enhances CXCR4-mediated migration of MDA-MB-231 cells

**Figure 6:**
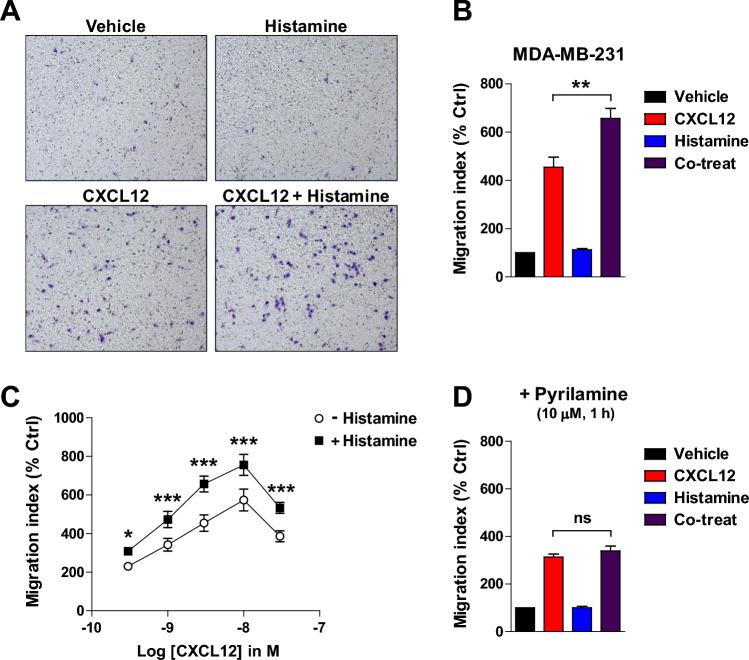
Costimulation of CXCR4 and HRH1 enhances CXCR4-mediated migration of MDA-MB-231 cells. (**A**) Representative images of MDA-MB-231 cells migrated by vehicle, 3 nM CXCL12, 100 nM histamine, or both agonists. (**B**) Quantification of migration shown in (**A**). Data are expressed as mean ± s.e.m. (n = 3). **P < 0.01. (**C**) CXCL12-induced cell migration in the absence or presence of 100 nM histamine. *P < 0.05; ***P < 0.001. (**D**) Cells were treated with 10 μM pyrilamine for 1 h before the migration assay with 3 nM CXCL12, 100 nM histamine, or both. Data are expressed as mean ± s.e.m. (n = 3). *Ns* not significant.

CXCL12/CXCR4-mediated cell migration plays an important role in cancer metastasis to distant organs and in drug resistance by mobilizing cancer cells to niches rich in survival-promoting factors^14,20,48–50^. Therefore, we investigated whether costimulation of CXCR4 and HRH1 affects CXCL12/CXCR4-mediated cell migration of MDA-MB-231 cells using a transwell migration assay. As expected, CXCL12 (3 nM) induced MB-231 cell migration, while histamine (100 nM) did not (Fig. 6A,B). Notably, however, histamine significantly enhanced CXCL12-induced migration. CXCL12 induced MDA-MB-231 cell migration in a dose-dependent manner with maximal migration at 10 nM (Fig. 6C). The addition of 100 nM histamine significantly increased CXCL12-induced migration at all CXCL12 doses tested. When cells were pretreated with the HRH1 antagonist pyrilamine, the effect of histamine on CXCL12-induced migration was abolished (Fig. 6D), demonstrating that the effect of histamine is mediated by HRH1.

**Figure 7:**
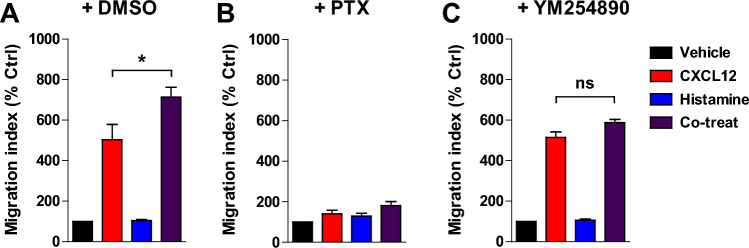
Gα_i/o_ plays a fundamental role in CXCR4-mediated migration and Gα_q/11_ is responsible for enhanced cell migration induced by costimulation of CXCR4 and HRH1. Cells were treated with vehicle (**A**), PTX (100 ng/ml, overnight) (**B**), or YM254890 (300 nM, 30 min) (**C**) before the migration assay with 3 nM CXCL12, 100 nM histamine, or both. Data are expressed as mean ± s.e.m. (n = 3). *P < 0.05; ns, not significant.

Next, to understand the signaling pathways involved in enhanced cell migration by cotreatment with CXCL12 and histamine, we investigated the effects of the Gα_i/o_ inhibitor PTX and the Gα_q/11_ inhibitor YM254890 on MDA-MB-231 cell migration. Consistent with previous reports that Gα_i/o_ signaling is important for CXCL12-induced migration^51,52^, PTX completely inhibited not only CXCL12-induced migration but also histamine-enhanced CXCL12-induced migration (Fig. 7A,B). In contrast, YM254890 selectively abrogated histamine-enhanced CXCL12-induced migration (Fig. 7C), suggesting that Gα_q/11_ activation is required for histamine-enhanced CXCL12-induced migration. These results suggest that the Gαi_i/o_ and Gα_q/11_ pathways, which are shown above to be important for calcium signaling, also play major roles in enhanced CXCR4-mediated cell migration upon costimulation of CXCR4 and HRH1.

### The synergistic effect of CXCR4 and HRH1 on calcium signaling and cell migration is observed in other cancer cell lines

**Figure 8:**
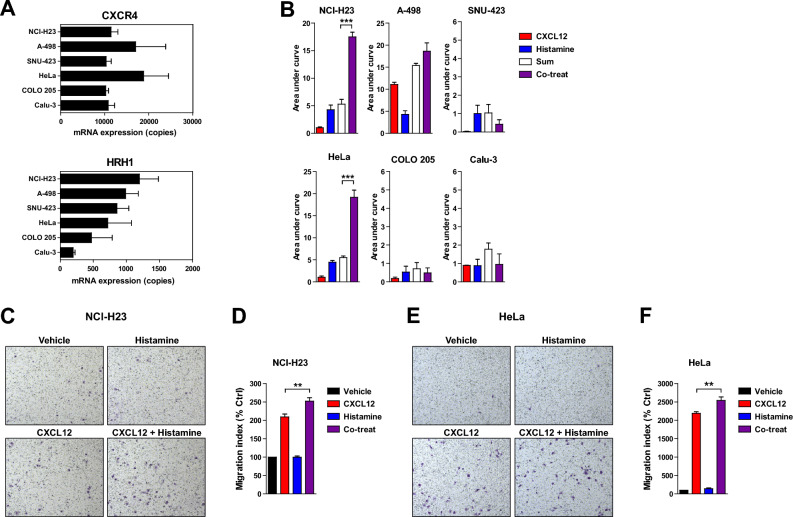
The synergistic effect of CXCR4 and HRH1 on calcium signaling and cell migration has been observed in other cancer cell lines. (**A**) RT‒qPCR analysis of the expression of CXCR4 (upper panel) and HRH1 (lower panel) in NCI-H23, A-498, SNU-423, HeLa, COLO 205, and Calu-3 cells. Data are expressed as mean ± s.e.m. (n = 3). (**B**) Cells were stimulated with 3 nM CXCL12, 100 nM histamine, or both, and intracellular calcium flux was measured. The area under the curve of each calcium response was calculated and normalized to that of CXCL12. Data are expressed as mean ± s.e.m. (n = 3). ***P < 0.001. (**C**) Representative images of NCI-H23 cells migrated by vehicle, 10 nM CXCL12, 100 nM histamine, or both agonists. (**D**) Quantification of migration shown in (**C**). Data are expressed as mean ± s.e.m. (n = 3). **P < 0.01. (**E**) Representative images of HeLa cells migrated by vehicle, 10 nM CXCL12, 100 nM histamine, or both agonists. (**F**) Quantification of migration shown in (**E**). Data are expressed as mean ± s.e.m. (n = 3). **P < 0.01.

To investigate whether enhanced calcium signaling and cell migration by concurrent treatment with CXCL12 and histamine are conserved in other cancer cell lines, we measured the expression of CXCR4 and HRH1 using RT-qPCR in NCI-H23 lung cancer cells, A-498 renal cancer cells, SNU-423 hepatocellular carcinoma cells, HeLa cervical cancer cells, COLO 205 colon cancer cells, and Calu-3 lung cancer cells. CXCR4 was highly expressed in all cell lines examined (Fig. 8A, upper panel). The expression of HRH1 was also high in NCI-H23, A-498, SNU-423, and HeLa cells, but was relatively low in COLO 205, and Calu-3 cells (Fig. 8A, lower panel).

Next, we examined calcium signaling and cell migration in these cancer cell lines. CXCL12 induced calcium flux in most cancer cell lines except SNU-423 cells (Fig. 8B), although SNU-423 cells showed similar levels of CXCR4 expression to NCI-H23, COLO 205, and Calu-3 cells. Histamine induced calcium flux in all cells. Notably, synergistic calcium flux was observed in NCI-H23, and HeLa cells. Intriguingly, while A-498 cells responded to CXCL12 with robust calcium flux, they did not show synergistic calcium flux upon simultaneous treatment with CXCL12 and histamine. It seems likely that calcium flux and its modulation are influenced by the cellular context, including membrane lipid composition and differential expression of receptor-interacting proteins, such as G protein subtypes, GPCR kinases, and PLCβ isoforms. NCI-H23 and HeLa cells also exhibited significantly enhanced cell migration upon simultaneous treatment with CXCL12 and histamine (Fig. 8C–F). Taken together, these results suggest that the synergistic effect of CXCR4 and HRH1 costimulation on calcium signaling and cell migration could be shared by several cancer cell lines.

In conclusion, our findings provide new insights into the role of HRH1 in the CXCL12/CXCR4 signaling pathway. CXCL12 is constitutively expressed by cancer-associated fibroblasts in the tumor microenvironment^14^, binds to CXCR4-expressing tumor cells, and mediates several cancer phenotypes, including proliferation, epithelial to mesenchymal transition, neoangiogenesis, and tumor cell migration^21^. In contrast, the roles of histamine and HRH1 in cancer pathology are controversial. Histamine either inhibits or stimulates cancerous phenotypes in a dose-dependent manner^53,54^. HRH1 also has both anticancer^32,55,56^ and procancer effects^33,57,58^. These controversies suggest that HRH1 does not function alone but may crosstalk with other signaling pathways. In the present study, we demonstrate that HRH1 physically interacts with CXCR4 and that the histamine/HRH1 pathway is functionally related to the CXCL12/CXCR4 pathway. Given that histamine is ubiquitously present in various tissues^59^, it is highly probable that CXCR4 and HRH1 in tumor cells that express both GPCRs can be simultaneously stimulated. Once coactivated, CXCR4 and HRH1 may contribute to tumor progression by enhancing calcium signaling and cell migration. Therefore, our findings suggest that the CXCR4-HRH1 heteromer may serve as a potential therapeutic target for anticancer therapy. Because antihistamines are commonly prescribed drugs and their safety has been proven for a long time, it will be worth investigating whether antihistamines can be repositioned for cancer treatment.

## Materials and methods

### Cell culture and reagents

MDA-MB-231 cells were purchased from the American Type Culture Collection (Rockville, MD). NCI-H23, A-498, SNU-423, HeLa, COLO 205, and Calu-3 cells were purchased from the Korean Cell Line Bank (Seoul, Korea). HEK293A cells were purchased from Invitrogen (Carlsbad, CA). MDA-MB-231, NCI-H23, A-498, SNU-423, and COLO 205 cells were cultured in RPMI 1640 supplemented with 10% fetal bovine serum (FBS), 100 U/mL penicillin, and 100 μg/mL streptomycin at 37 °C in 5% CO_2_. HeLa cells were grown in modified Eagle’s medium (MEM; HyClone, Logan, UT) supplemented with 10% FBS, penicillin, and streptomycin. HEK293A and Calu-3 cells were cultured in Dulbecco’s modified Eagle’s medium (DMEM; HyClone) supplemented with 10% FBS, penicillin, and streptomycin. CXCL12 (#300-28A) was purchased from PeproTech (London, UK). Histamine (#H7250) was purchased from Sigma‒Aldrich (St. Louis, MO). AMD3100 (#HY10046), U73122 (#HY-13419), and U0126 (#HY-12031) were purchased from MedChemExpress (Monmouth Junction, NJ). Pyrilamine (#0660) and PTX (#3097) were purchased from Tocris Bioscience (Ellisville, MO). YM254890 (#AG-CN2-0509-MC05) was purchased from AdipoGen Life Sciences (San Diego, CA).

### BiFC assay

The bimolecular fluorescence complementation (BiFC) assay is a technique to detect protein–protein interactions in living cells in which two complementary N- and C-terminal fragments of fluorescent protein reconstitute a fluorescent signal only when both fragments are close together through interaction between the two different proteins to which they are fused^35^. Vectors containing the BiFC fragments (pCS2 + VNm10 and pBiFC-VC155) were obtained from James Smith^60^ and Chang-Deng Hu^35^. The complementary fragments of Venus protein (VNm10, VC155) were subcloned at the C-termini of CXCR4, HRH1, and OPRM1. For the BiFC experiments, HEK293A cells (1 × 10^4^ cells/well) were seeded onto a 96-well black clear-bottom microplate (#3340) purchased from Corning (Corning, NY). The next day, cells were cotransfected with complementation pairs (CXCR4-VN/CXCR4-VC, CXCR4-VN/HRH1-VC, HRH1-VN/CXCR4-VC, CXCR4-VN/HA-OPRM1-VC, or HA-OPRM1-VN/CXCR4-VC). 48 h after transfection, the cells were fixed with 2% formaldehyde and stained with Hoechst 33342 (Invitrogen). Rabbit anti-HA monoclonal antibody (#3724, Cell Signaling Technology, Beverly, MA) was used to detect the cell surface expression of N-terminal HA-tagged OPRM1-VN and -VC constructs. Briefly, fixed cells were blocked in DPBS containing 1% BSA and immunostained overnight at 4 °C. Cells were washed twice and labeled with goat anti-rabbit IgG-Alexa Fluor 568 antibody (#A11011, Invitrogen) for 1 h at room temperature. Cells were washed twice after secondary antibody incubation, and BiFC, cell surface immunofluorescence, and nuclear images were captured using a Zeiss LSM 700–2 confocal microscope (40 × objective).

### BRET donor saturation assay

In the BRET donor saturation assay, a fixed concentration of bioluminescent-tagged donor protein and variable concentrations of fluorescent-tagged acceptor protein are expressed in cells. The interaction between two proteins can be examined by quantifying the dependence of the BRET signal on the acceptor/donor expression ratio^38,39^. HEK293A cells were seeded onto 24-well plates. The next day, cells were transfected with the BRET donor (3, 10, 30, 100 ng of GPCR-Rluc8) and the BRET acceptor (0, 10, 30, 100, 300 ng of GPCR-mCitrine). Transfections were performed using Lipofectamine 2000 (Invitrogen). The total amount of transfected DNA was fixed to 400 ng using empty vector. 24 h after transfection, the cells were detached in assay buffer (DMEM without phenol red, 20 mM HEPES) and distributed onto 96-well white microplates (#3917; 4 × 10^4^ cells/well) purchased from Corning. The next day, acceptor expression in each cells were measured with excitation 485/14, emission 535/25 using Tristar2 microplate reader (Berthold Technologies, Bad Wildbad, Germany). Next, the Rluc8 substrate coelenterazine h (NanoLight Technology, Pinetop, AZ) was added at a final concentration of 5 μM. Cells were further incubated for an additional 5 min before BRET measurements. BRET measurements were also performed using a TriStar2 microplate reader. The acceptor/donor ratios of each transfected cells were calculated by dividing mCitrine relative fluorescence unit (RFU) by Rluc8 relative luminescence unit (RLU).

### Gene knockout using the CRISPR/Cas9 system

Nontargeting single guide RNA (sgControl; 5′-ACGGAGGCTAAGCGTCGCAA-3′), CXCR4-targeting single guide RNA (sgCXCR4; 5′-ACTTACACTGATCCCCTCCA-3′), or HRH1-targeting single guide RNA (sgHRH1; 5′-CGATCAAGTCCGCCACCGAG-3′) cloned in pLentiCRISPRv2 vectors were purchased from Genscript (Piscataway, NJ). The pLentiCRISPRv2 plasmid for each single guide RNA was cotransfected into HEK293T cells with packaging plasmids for lentiviral production. MDA-MB-231 cells transduced with lentiviruses containing sgControl, sgCXCR4, or sgHRH1 were cultured for 2 weeks with puromycin selection (1 μg/ml). Knockout of CXCR4 or HRH1 genes was validated using flow cytometry and calcium flux assays.

### Calcium flux assay

Intracellular calcium mobilization was measured using Cal-520 AM calcium staining dye (AAT Bioquest, Sunnyvale, CA) according to the manufacturer’s directions. Briefly, MDA-MB-231 cells (4 × 10^4^ cells/well) were seeded into a 96-well black clear-bottom microplate (#3340, Corning). After 48 h, the cells were stained with Cal-520 AM dye diluted in assay buffer (HBSS, 0.1% BSA, 20 mM HEPES) for 2 h at 37 °C. After staining, the cells were washed with assay buffer, and inhibitors or vehicle were pretreated for 30 min. In the case of PTX pretreatment, the cells were pretreated overnight the day before staining. Intracellular calcium flux was measured by a Flexstation 3 microplate reader (Molecular Devices, San Jose, CA).

### cAMP signaling assay

Intracellular cAMP signaling was measured using the GloSensor-22F cAMP reporter (Promega, Madison, WI). MDA-MB-231 cells transduced with lentiviruses containing the GloSensor-22F were cultured for 2 weeks with hygromycin selection (200 μg/ml). The day before experiments, MDA-MB-231 cells expressing the biosensor were seeded into a 96-well white microplate (#3917, Corning) and incubated at 37 °C and 5% CO_2_. The next day, the culture medium was replaced with assay buffer containing 0.1% BSA and 2% Glosensor cAMP reagent in CO_2_-independent medium (Gibco, Gaithersburg, MD). After 2 h of incubation at room temperature, intracellular cAMP was measured using a TriStar2 microplate reader.

### RT‒qPCR

Total RNA from cells was isolated using the RNeasy Mini Kit (Qiagen, Valencia, CA). First-strand cDNA was synthesized using the ReverTra Ace qPCR RT kit (#FSQ-101, Toyobo, Osaka, Japan). RT-qPCR was performed by using Brilliant SYBR Green QPCR Master Mix (Agilent Technologies, Palo Alto, CA), and the following PCR conditions were used: 95 °C for 3 min, followed by 40 cycles of 95 °C for 5 s and 60 °C for 30 s, and followed by 95 °C for 15 s and 60 °C for 60 s melt curve analysis to check amplification specificity, using Quantstudio3 instrument (Applied Biosystems, Foster City, CA). The absolute level of each GPCR cDNA was measured by using a standard curve generated with the GPCR plasmid template and gene-specific primers. The primer sequences used are: CXCR4: 5′-CCACCATCTACTCCATCATCTTC-3′ and 5′-ACTTGTCCGTCATGCTTCTC-3′; HRH1: 5′-CCTCTGCTGGATCCCTTATTTC-3′ and 5′-GGTTCAGTGTGGAGTTGATGTA-3′; HRH2: 5′-AGTGCAAAGTCCAGGTCAAT-3′ and 5′-GAAGATGCGGTAGTAGGTGATG-3′; HRH3: 5′-CTGCTATGCCGAGTTCTTCTAC-3′ and 5′-GATGTTCAGGTAGATGCTGAGG-3′; HRH4: 5′-TCCTTGCCATCACATCATTCT-3′ and 5′-CTACTGAGATGATCACGCTTCC-3′; and GAPDH: 5′-ATGACATCAAGAAGGTGGTGAA-3′ and 5′-GCTGTTGAAGTCAGAGGAGAC-3′.

### Transwell migration assay

Cell migration was assayed in 24-well cell culture plates using transwell inserts with 8 μm pore membranes (#3422, Coring). Transwell inserts were precoated with collagen I (50 μg/mL). MDA-MB-231 cells were seeded at 80% confluency in 60 mm cell culture dishes. The next day, the cells were serum-starved overnight. Then, the cells were detached and resuspended (1 or 2 × 10^5^ cells/mL) in assay buffer (0.5% BSA in RPMI 1640). After incubation for 3 h, cells on the lower surface of the transwell inserts were fixed with 4% paraformaldehyde and stained with crystal violet. Stained cells were counted in at least 10 different fields under light microscopy (× 200 magnification).

### Flow cytometry

Cells were detached using PBS containing 10 mM EDTA and labeled with mouse anti-CXCR4 primary antibody (#4G10, Santa Cruz Biotechnology, Santa Cruz, CA), mouse anti-HRH1 monoclonal antibody provided by Dr. Hosun Son (GPCR Therapeutics, Seoul, Korea), or rabbit anti-HA monoclonal antibody (#3724, Cell Signaling Technology). An APC-conjugated goat anti-mouse IgG antibody (R&D Systems, Abingdon, UK) was used to label CXCR4 and HRH1, and goat anti-rabbit IgG-Alexa Fluor 488 antibody (#A32731, Invitrogen) was used to label HA tag. Cells were analyzed with a FACS Canto II (BD Biosciences, San Jose, CA).

### Expression and survival analysis

To evaluate the association between CXCR4 and HRH1 expression and prognosis in breast cancer, publicly available datasets from TCGA database were analyzed. The expression and clinical data of TCGA Breast Invasive Carcinoma (BRCA) were downloaded from the UCSC Xena Browser (https://xenabrowser.net/). Both violin plots for expression data and Kaplan–Meier survival curves were analyzed using R version 4.1.2 (http://www.r-project.org/).

### Statistical analysis

Statistical significance was determined by one-way ANOVA followed by Tukey’s multiple comparison test, and two-way ANOVA followed by Bonferroni’s multiple comparison test using GraphPad Prism software (GraphPad Software, San Diego, CA).

### Supplementary Information

Supplementary Information.

## Data Availability

Cancer cell line RNA-seq data were downloaded from the CCLE (https://sites.broadinstitute.org/ccle/). The expression and clinical data of TCGA were downloaded from the UCSC Xena Browser (https://xenabrowser.net/). All data generated or analyzed during this study are included in this published article and its Supplementary Information files.
