# Author Correction: Simultaneous activation of CXC chemokine receptor 4 and histamine receptor H1 enhances calcium signaling and cancer cell migration

**DOI:** 10.1038/s41598-024-60108-4

**Published:** 2024-04-24

**Authors:** Chulo Park, Jin‑Woo Lee, Kiheon Kim, Dong‑Seung Seen, Jae‑Yeon Jeong, Won‑Ki Huh

**Affiliations:** 1 School of Biological Sciences, Seoul National University, Seoul, 08826 Republic of Korea; 2 GPCR Therapeutics Inc., Gwanak-gu, Seoul, 08790 Republic of Korea; 3 Institute of Microbiology, Seoul National University, Seoul, 08826 Republic of Korea

Correction to: *Scientific Reports* 10.1038/s41598-023-28531-1, published online 02 February 2023

The original version of this Article omitted an affiliation for Won-Ki Huh. The correct affiliations are listed below.

School of Biological Sciences, Seoul National University, Seoul, 08826, Republic of Korea.

Institute of Microbiology, Seoul National University, Seoul, 08826, Republic of Korea.

GPCR Therapeutics Inc., Gwanak-gu, Seoul 08790, Republic of Korea.

The original Article has been corrected.

