# Supplementary material for: Simultaneous activation of CXC chemokine receptor 4 and histamine receptor H1 enhances calcium signaling and cancer cell migration
Source: Sci Rep. 2023 Feb 2;13:1894. doi: 10.1038/s41598-023-28531-1 (PMC9895059; doi:10.1038/s41598-023-28531-1)
Supplement: Supplementary file 1 — Supplementary Information. [file 41598_2023_28531_MOESM1_ESM.docx]

**SUPPLEMENTARY INFORMATION**

**Simultaneous activation of CXC chemokine receptor 4 and histamine receptor H1 enhances calcium signaling and cancer cell migration**

**Chulo Park^1,2^, Jin-Woo Lee^1^, Kiheon Kim^1^, Dong-Seung Seen^2^, Jae-Yeon Jeong^2,^* & Won-Ki Huh^1,3,^***

^1^School of Biological Sciences, Seoul National University, Seoul 08826, Republic of Korea

^2^GPCR Therapeutics Inc., Gwanak-gu, Seoul 08790, Republic of Korea

^3^Institute of Microbiology, Seoul National University, Seoul 08826, Republic of Korea

*Correspondence: [jeongjy@gpcr.co.kr](mailto:jeongjy@gpcr.co.kr); [wkh@snu.ac.kr](mailto:wkh@snu.ac.kr)

**Supplementary Tables**

**Table S1.** Analysis of CCLE cancer cell lines coexpressing CXCR4 and HRH1. Cancer cell lines expressing both CXCR4 and HRH1 higher than 5 TPM were counted as coexpressing cell lines.

| Cancer Type | No. of cell lines | No. of cell lines co-expressing CXCR4 and HRH1 | % of cell lines co-expressing CXCR4 and HRH1 |
| --- | --- | --- | --- |
| Astrocytoma | 9 | 3 | 33% |
| Bile duct cancer | 6 | 3 | 50% |
| Bladder carcinoma | 25 | 2 | 8% |
| Breast cancer | 55 | 6 | 11% |
| Cecum adenocarcinoma | 8 | 1 | 13% |
| Colon cancer | 41 | 10 | 24% |
| Colorectal cancer | 10 | 0 | 0% |
| Esophageal cancer | 26 | 2 | 8% |
| Gastric cancer | 40 | 4 | 10% |
| Glioblastoma | 31 | 4 | 13% |
| Hepatic cancer | 23 | 3 | 13% |
| Leukemia | 81 | 4 | 5% |
| Lung cancer | 175 | 12 | 7% |
| Lymphoma | 55 | 4 | 7% |
| Medulloblastoma | 4 | 1 | 25% |
| Melanoma | 53 | 0 | 0% |
| Mesothelioma | 11 | 2 | 18% |
| Multiple Myeloma | 24 | 0 | 0% |
| Neuroblastoma | 15 | 2 | 13% |
| Ovarian cancer | 47 | 9 | 19% |
| Pancreatic cancer | 41 | 6 | 15% |
| Prostate cancer | 7 | 0 | 0% |
| Renal cancer | 23 | 7 | 30% |
| Sarcoma | 38 | 2 | 5% |
| Thyroid cancer | 11 | 5 | 45% |
| Uterine cancer | 30 | 7 | 23% |
| Other cancers/tumors | 45 | 0 | 0% |
| Total­­ | 934 | 99 | 11% |

**Supplementary Figures**


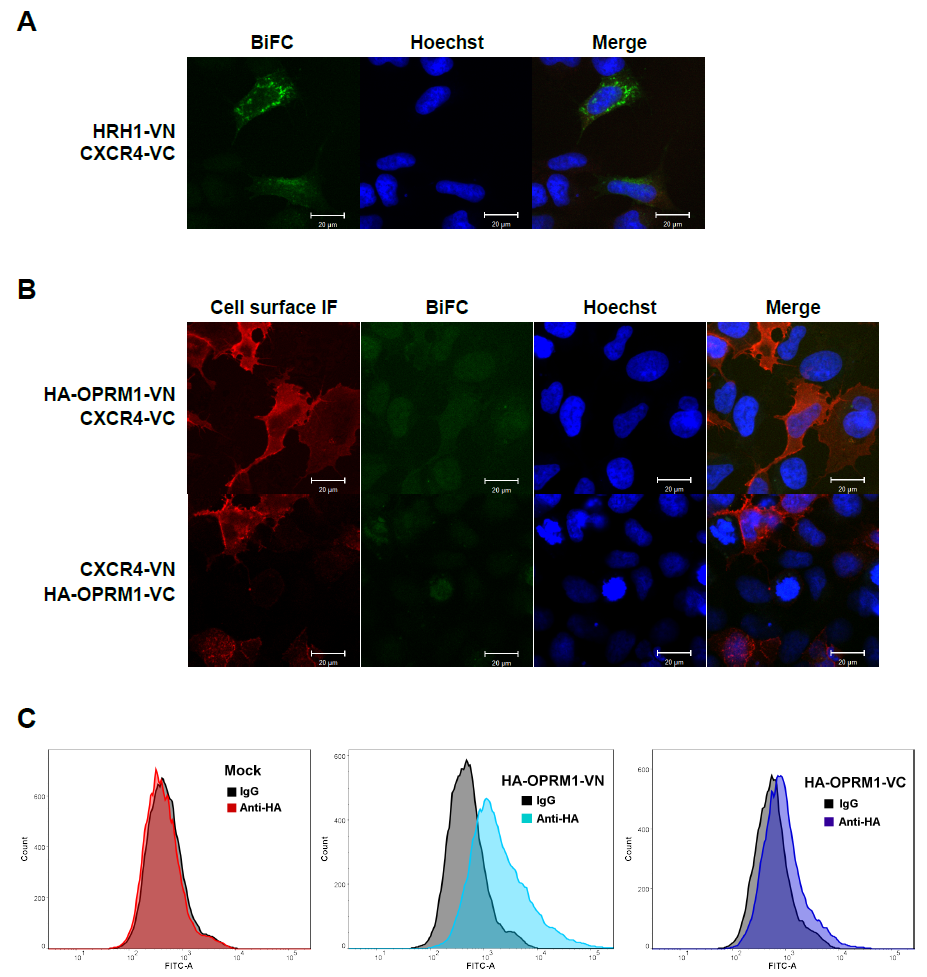


**Figure S1.** (**A,B**) Analysis of heteromerization between CXCR4 and HRH1 or CXCR4 and OPRM1 using the BiFC assay. HEK293A cells were transfected with HRH1-VN and CXCR4-VC (**A**), HA-OPRM1-VN and CXCR4-VC (**B**, upper panel), or CXCR4-VN and HA-OPRM1-VC (**B**, lower panel). To detect the cell surface expression of HA-OPRM1-VN and HA-OPRM-VC, cells were stained with rabbit anti-HA monoclonal antibody and goat anti-rabbit IgG-Alexa Fluor 568 antibody without permeabilization. Cell nuclei were stained with Hoechst 33342. Images are representative of three independent experiments. (**C**) Flow cytometric analysis of the cell surface expression of HA-OPRM1-VN and HA-OPRM-VC. HEK293A cells transfected with either HA-OPRM1-VN or HA-OPRM1-VC constructs, or empty vector. The N-terminal HA epitope was detected with rabbit anti-HA monoclonal antibody and goat anti-rabbit IgG-Alexa Fluor 488 antibody without permeabilization. Images are representative of three independent experiments.


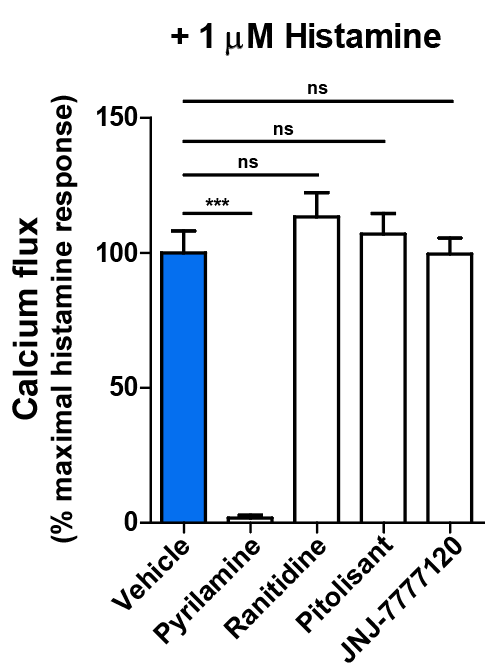


**Figure S2.** Effects of histamine receptor antagonists on histamine-induced calcium flux in MDA-MB-231 cells. Cells were pretreated with the HRH1 antagonist pyrilamine, the HRH2 antagonist ranitidine, the HRH3 antagonist pitolisant, or the HRH4 antagonist JNJ-7777120 (10 μM each) for 30 min and stimulated with 1 μM histamine. Intracellular calcium flux was measured using CAL520-AM. Data are expressed as mean ± s.e.m. (n=3). ****P* < 0.001; ns, not significant.


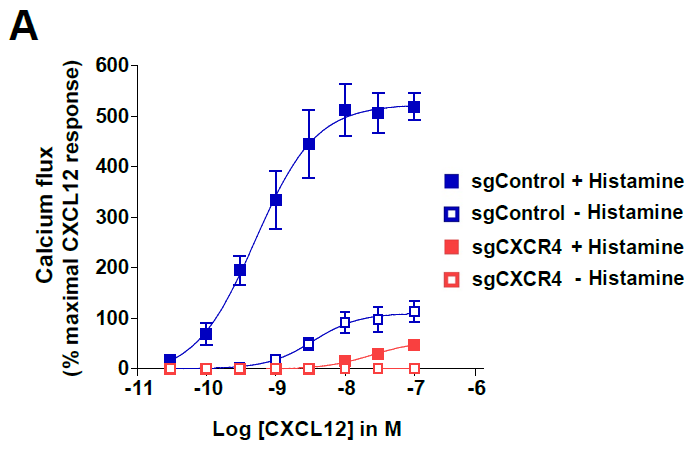


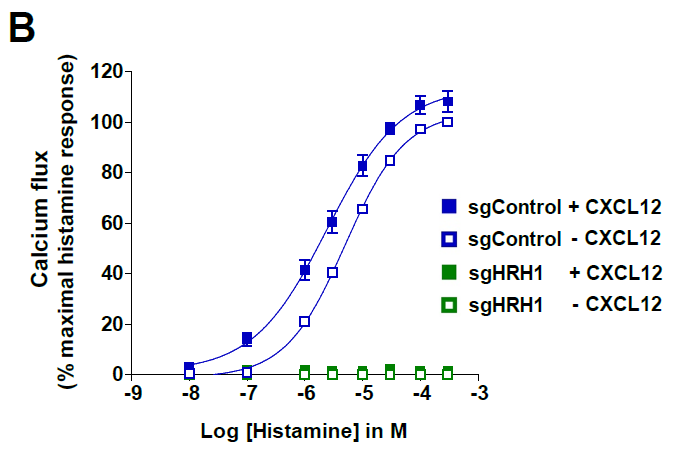


**Figure S3.** Synergistic calcium flux is mediated by CXCR4 and HRH1. (**A**) Intracellular calcium flux was measured by stimulating CXCR4 knockdown (sgCXCR4) or control (sgControl) cells with increasing concentrations of CXCL12 in the absence or presence of 100 nM histamine. (**B**) Intracellular calcium flux was measured by stimulating HRH1 knockdown (sgHRH1) or control (sgControl) cells with increasing concentrations of histamine in the absence or presence of 3 nM CXCL12. Data are expressed as mean ± s.e.m. (n = 3).


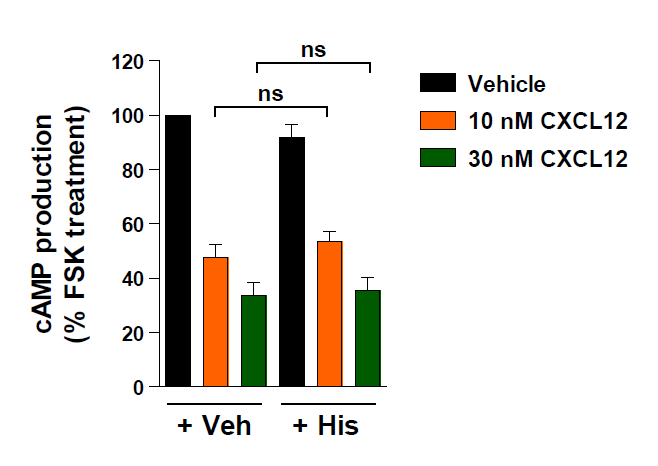


**Figure S4.** Costimulation of CXCR4 and HRH1 does not affect CXCR4-mediated cAMP signaling in MDA-MB-231 cells. Cells were pretreated with forskolin and the effect of CXCL12 on cAMP production was measured in the absence or presence of 100 nM histamine. Data are expressed as mean ± s.e.m. (n = 3). ns, not significant.
